# Weekly Trends in Preschoolers’ Physical Activity and Sedentary Time in Childcare

**DOI:** 10.3390/ijerph120302454

**Published:** 2015-02-24

**Authors:** Leigh M. Vanderloo, Patricia Tucker

**Affiliations:** 1 Health and Rehabilitation Sciences, Faculty of Health Sciences, University of Western Ontario, London, ON N6G 1H1, Canada; E-Mail: lvande32@uwo.ca; 2 School of Occupational Therapy, Faculty of Health Sciences, University of Western Ontario, London, ON N6G 1H1, Canada

**Keywords:** preschooler, physical activity, sedentary time, childcare, accelerometer

## Abstract

This study sought to examine how the physical activity levels and sedentary time of preschoolers attending center-based childcare varied across the week. Sex differences were also explored. Participants (*n* = 101) wore Actical™ accelerometers (15 s epoch) for five consecutive days during childcare hours only. A multivariate repeated measures analysis of variance was used to evaluate levels of sedentary, light, and moderate-to-vigorous (MVPA) physical activity across the five weekdays. Total physical activity (TPA) was analyzed separately in a univariate repeated measures ANOVA. Sex was entered as an additional between-subjects factor. Levels of sedentary time, LPA, and TPA across the week were found to be statistically significant, and can best be described by quadratic effects. Participants’ activity levels and sedentary time typically peaked mid-week. Levels of physical activity and sedentary time were not found to significantly differ based on sex. Childcare centers may benefit from the introduction and/or modification of active play-based programming and curricula, particularly at the start and end of the week where preschoolers’ activity levels tend to be lower. Additional investigations are required to confirm these findings.

## 1. Introduction

Regular physical activity among young children has been linked to a plethora of health benefits [1]. Likewise, low levels of sedentary behaviors have been noted in the literature as preventing many chronic conditions [2]. In light of recent research which suggests that activity levels are low [3,4,5] and sedentary behaviors high [3,6,7] among this cohort, the preschool period (*i.e.*, 2.5–5 years) may serve as an ideal time to minimize sedentary behaviors and promote physical activity.

A large proportion of Canadian preschoolers attend some form of non-parental care [8], with the majority being enrolled in center-based childcare. Benjamin and colleagues highlight the childcare environment as a unique setting in which young children can establish many health-related behaviors [9], inclusive of physical activity and sedentary pursuits. However, despite the potential of this setting to encourage active behaviors and reduce sedentary ones, research suggests that this is not the case [3,10,11,12]. Recent qualitative work by Tucker *et al*. [13,14] and van Zandvoort *et al*. [15] have highlighted a number of challenges experienced by childcare providers while trying to promote physical activity participation among the children in their care. In fact, childcare providers have noted that children’s activity levels vary over the course of the week, which in and of itself, serves as a barrier to maintaining active behaviors during care hours [14]. Further exploration into young children’s weekly activity trends in childcare is warranted as a means of assisting childcare providers to re-configure current daily programming in support of increasing active behaviors and minimizing sedentary ones.

A gold standard in the objective measurement of preschoolers’ physical activity and sedentary behaviors [16,17], accelerometers provide researchers with useful data including the intensity, frequency, duration, and time (*i.e.*, day of the week; hour of the day) at which the activity occurred. Most research to date has explored *average* daily physical and sedentary levels among preschoolers [7,18]; activity levels during outdoor play periods [19,20] and childcare [3,11,12,21,22,23], as well as hourly [24,25] and seasonal patterns [26,27,28] of preschoolers’ activity. Despite the utility of accelerometers to provide detailed information on day-by-day patterning of preschoolers’ activity levels, no studies have examined this occurrence among young children while attending childcare.

The purpose of this study was to explore how the physical activity levels and sedentary time of preschoolers in center-based childcare varied over the course of the week. Sex differences across the days of the week were also explored. In accordance with previously published work [14], it is hypothesized that preschoolers will be most sedentary and least active at the start of the week, with physical activity levels increasing over the course of the week. This is the first study to investigate this relationship, which is important given that childcare staff may need to modify their programming and curriculum in order to support activity differently throughout the week.

## 2. Material and Methods

### 2.1. Study Design and Participants

This project was part of a larger 2-year cross-sectional study, the *Learning Environments’ Activity Potential for Preschoolers* (LEAPP). Specifics regarding the methodology have been articulated elsewhere [29]. Because only staff from center-based childcare (and not home-based childcare or kindergarten programs) have reported the daily variation in activity behaviors among preschoolers, for the purpose of this paper, only the subset of preschool children (*i.e.*, 2.5–5 years) enrolled in center-based childcare in London, Ontario, Canada were examined [14]. Preschoolers from nine childcare centers were included in this sample. Children who received parental/guardian consent were eligible to participate in the study. Ethical approval was obtained from the University of Western Ontario’s Research Ethics Board for all study material and protocol.

### 2.2. Data Collection

Data collection took place from September 2011 to June 2012. Preschoolers’ activity levels were assessed objectively using Actical™ (MiniMitter, Bend, Oregon) accelerometers, at a 15 second time sampling interval. Participants were asked to wear the devices for five consecutive days during childcare hours only (as only participants’ activity/sedentary levels within this environment were of interest); specifically, childcare staff were asked to place the devices on preschoolers’ right hip with an elastic waistband upon arrival at childcare in the morning, and to remove them at end-of-day prior to the children leaving for home. Staff were also asked to keep a wear-time log for each of the preschool participants (*i.e.*, to note the on/off times that each individual children wore the accelerometer). Researchers returned to the participating childcare centers to retrieve all study material following data collection (*i.e.*, at the end of the five day monitoring period).

### 2.3. Statistical Analysis

Accelerometry data was downloaded using Actical-specific software. *KineSoft* version 3.3.62 (KineSoft, Saskatchewan, Canada; a custom software program) was used to clean the Actical data and to help run reliability analyses. Specifically, participants with at least one valid day of wear-time were included in the analyses (where a minimum of five hours of wear-time constituted a valid day); these inclusion criteria were based on previous work carried out by Colley and colleagues [30]. Past research in the childcare environment by Vanderloo *et al*. [3] also included participants with one valid day of data. Non-wear-time was defined as 60 minutes of consecutive zeros [30,31]. Pfeiffer and colleagues’ preschooler-specific cut-points were applied to the accelerometer data (*i.e.*, sedentary [<50 counts per 15 second epoch], light physical activity [LPA; ≥50 ≤714 counts per 15 second epoch], and moderate-to-vigorous physical activity [MVPA; ≥715 counts per 15 second epoch]) [16]. Physical activity and sedentary time per hour of wear time was calculated to account for the varying lengths of time participants spent in childcare. Percentages of monitoring time spent at the various intensity levels of interest were also calculated. All statistical tests were carried out using SPSS version 22. For the variables sedentary, LPA, and MVPA, we conducted a multivariate repeated measures analysis of variance (MANOVA), in which the days of the week served as the repeated measure. Due to the fact that total physical activity (TPA) is a calculated variable that is a linear combination of LPA and MVPA, it was analyzed separately, in a univariate repeated measures analysis of variance (ANOVA). As a means of exploring sex differences in preschoolers’ physical activity levels and sedentary time between days of the week, sex was added as an additional between-subjects factor.

## 3. Results

The majority of preschool participants (*n* = 101, mean age = 3.55 years [*SD* = 0.90]) were Caucasian (79.2%) and 56.4% of the sample was female.

### 3.1. Physical Activity and Sedentary Time across the Week

**Figure 1:**
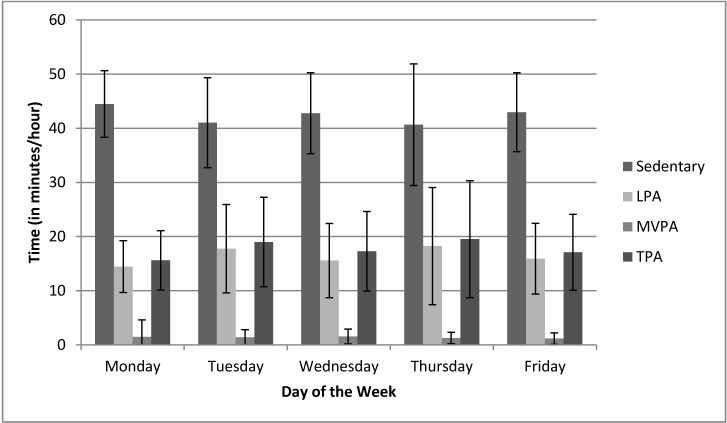
Physical activity and sedentary time based on day of the week, during childcare hours.

Notes: LPA = light physical activity; MVPA = moderate-to-vigorous physical activity; TPA = total physical activity (light, moderate, and vigorous activity).

The canonical variate created from the optimally weighted combination of sedentary time, LPA, and MVPA was statistically significant (*F*[12,1152] = 2.12, *p* = 0.01), suggesting that the univariate analyses for sedentary time, LPA, and MVPA may be interpreted without adjusting the per-comparison alpha [32]. The univariate analysis for sedentary time was statistically significant (*F*[3.74,323.94] = 4.44, *p* = 0.003), as was the univariate analysis for LPA (*F*[3.18,304.94] = 5.17, *p* = 0.001). The univariate analyses for MVPA (*F*[1.74,166.69] = 0.94, *p* = 0.38) was not statistically significant. To evaluate the trend for each of these variables across the days of the week, we calculated polynomial contrasts for each variable, testing both linear and quadratic effects. Sedentary time and LPA were best described by a quadratic effect, *F*(1,96) = 10.84, *p* = 0.001 and *F*(1,96) = 9.38, *p* = 0.003, respectively. No contrasts were found to be statistically significant for MVPA. Descriptives for each of these variables (including percentage of monitoring time spent at these intensities), separated by days of the week, are presented in Table 1. See Figure 1 for a visual depiction of the activity data.

**Table 1:** Preschoolers’ Rates (in minutes/hour) and Percentage (of wear time) of Physical Activity and Sedentary Time across the Days of Week during Childcare Hours

Intensity Levels		Monday		Tuesday		Wednesday		Thursday		Friday	
		*n* = 99		*n* = 101		*n* = 100		*n* = 101		*n* = 101	
		WT = 346.05 (153.04)		WT = 379.92 (162.31)		WT = 371.54 (159.55)		WT = 336.82 (185.91)		WT = 333.97 (172.60)	
		Mean (*SD*)	95% CI	Mean (*SD*)	95% CI	Mean (*SD*)	95% CI	Mean (*SD*)	95% CI	Mean (*SD*)	95% CI
SED	Rate	44.48 (6.16)	[43.27, 45.69]	41.03 (8.32)	[39.41, 42.65]	42.77 (7.48)	[41.30, 44.24]	40.65 (11.23)	[38.46, 42.84]	42.97 (7.28)	[41.55, 44.39]
	% of wear time	74.24 (10.27)	[72.77, 76.26]	68.54 (13.73)	[65.86, 71.22]	71.64 (12.60)	[69.17, 74.11]	67.08 (19.64)	[63.25, 70.91]	71.22 (13.27)	[69.80, 72.64]
LPA	Rate	14.45 (4.78)	[13.51, 15.39]	17.77 (8.17)	[10.18, 13.36]	15.75 (6.84)	[14.23, 16.91]	18.24 (10.81)	[16.13, 20.35]	15.91 (6.54)	[14.63, 17.19]
	% of wear time	23.99 (7.96)	[22.42, 25.56]	29.49 (13.48)	[26.86, 32.12]	25.94 (11.50)	[23.69, 28.19]	31.08 (19.07)	[27.36, 34.80]	26.98 (12.24)	[24.59, 29.37]
MVPA	Rate	1.48 (3.13)	[0.86, 2.10]	1.40 (1.41)	[1.13, 1.67]	1.54 (1.37)	[1.27, 1.81]	1.27 (1.06)	[1.06, 1.48]	1.17 (1.04)	[0.97, 1.37]
	% of wear time	2.45 (5.20)	[1.22, 3.26]	2.30 (2.32)	[1.85, 2.75]	2.52 (2.29)	[2.07, 2.97]	2.10 (1.76)	[1.76, 2.44]	1.91 (1.74)	[0.97, 1.37]
TPA	Rate	15.61 (5.49)	[14.53, 16.69]	19.00 (8.26)	[17.39, 20.61]	17.29 (7.36)	[15.85, 18.73]	19.52 (10.79)	[17.42, 21.62]	17.10 (7.00)	[15.73, 18.47]
	% of wear time	25.92 (9.17)	[24.11, 27.73]	31.52 (13.63)	[28.86, 24.18]	28.45 (12.41)	[26.02, 30.88]	33.19 (18.95)	[29.52, 36.86]	28.90 (12.84)	[26.40, 31.40]

Notes: SED = sedentary time; LPA = light physical activity; MVPA = moderate-to-vigorous physical activity; TPA = total physical activity (light, moderate, vigorous activity); *SD* = standard deviation; CI = confidence interval; WT = average daily wear-time in minutes (*SD*).

The repeated measures ANOVA for TPA was also statistically significant (*F*(4,384) = 4.89, *p* = 0.002), and the polynomial contrast conducted on this variable suggested that the trend was best described by a quadratic effect (*F*(1,96) = 11.16, *p* = 0.001). Descriptives for this variable are also presented in Table 1.

### 3.2. Sex Differences in Physical Activity and Sedentary Time across the Week

No statistically significant differences were observed among levels of sedentary time (*F*[3.37,323.93] = 0.71, *p* = 0.56), LPA (*F*[3.18,304.94] = 1.21, *p* = 0.31), MVPA (*F*[1.74,6.35] = 1.62, *p* = 0.20), or TPA (*F*[3.40,326.75] = 1.09, *p* = 0.40) among male and female preschoolers across the week.

## 4. Discussion

The aim of this research was to examine how physical activity and sedentary time of preschoolers attending center-based childcare differed over the course of the week. The chief finding of this work suggests that preschoolers accumulated the most activity (*i.e.*, LPA, MVPA, and TPA) mid-week (*i.e.*, Tuesday to Thursday); specifically, participants’ activity levels were found to gradually increase at the beginning of the week, peak mid-week, and then begin to decrease again by the end of the week.

These findings were consistent with Tucker and colleagues’ qualitative work which suggested that young children’s activity levels vary based on the day of the week [14], thus serving as a barrier to maintaining active behaviors during childcare hours. Specifically, childcare providers have reported that “[on] Mondays they [the children] are very lethargic and [say] “I don’t want to. I’m bored. By the end of the week you can see the influences of the [childcare providers] …and the playing they’re doing [has] changed when they go home on the weekend. And [we] start all over again on Monday” (p. 4). While data which explores day of the week variations in preschoolers’ activity levels are lacking, there are studies to support the fact that preschoolers tend to be more active during the weekdays than on weekends [25,33], which may explain why the present sample’s total physical activity levels were lower at the start of the week. Although not specific to preschoolers in the childcare environment, a study by Telford and colleagues found that children’s (ages 8–12 years) *total* objectively measured physical activity levels increased over the course of the week, typically plateauing mid-week [34]; this suggests that similar trends exists among slightly older cohorts as well. Also of interest to note, while preschoolers’ LPA and TPA levels did decrease from Thursday to Friday, their activity levels were still slightly higher at the end of the week than at the start of the week. Such findings may be a function of the programming and instruction the children receive in childcare related to active play. In addition, given the influence of childcare staff on preschoolers’ physical activity levels [3], these trends in activity levels could also be a result of the providers’ own personal weekly activity behaviors (*i.e.*, their energy levels, level of motivation, *etc*.). It is also possible that childcare staff have different programming planned for the latter part of the week, which therefore results in less opportunity for active play. As for sex differences, it was somewhat surprising that sex did not appear to significantly impact the physical activity levels of the preschool participants over the course of the week; much published research to date reports male preschoolers accumulating more activity than their female counterparts [3,4]. While the authors are unsure as to why no differences in physical activity levels based on sex were observed, this non-significant finding could be attributed to the fact that the childcare environment may be prescriptive of young children’s activity behaviors. Additional queries examining this finding may be warranted.

With regard to preschoolers’ sedentary behaviors, it was found that the time spent by participants’ at this particular intensity level appeared to fluctuate throughout the week (*i.e.*, alternating high-low peaks); however, in general, sedentary time appeared to be lowest mid-week. This finding did not quite align with to what researchers expected to find, where it was hypothesized that preschoolers’ sedentary time would decrease over the course of the week. Moreover, it is also worth noting that levels of sedentary time loosely followed the physical activity trends observed in this study (*i.e.*, participants appeared to accumulate more activity and less sedentary time mid-week). One possible explanation for this trend may be a result of programming variations over the course of the week (*i.e.*, activities made available to preschoolers in care at that time of the week may have been more sedentary in nature—reading and crafts for example) as well as any changes in staff’s levels of motivation and personal activity behaviors mid-week.

This study sheds light on the variability of preschoolers’ physical activity levels and sedentary time within the childcare center. In light of the present findings, as well as current physical activity guidelines for the early years (*i.e.*, 180 minutes of daily activity at any intensity) [35], it is recommended that childcare centers tailor their daily programming to better support preschoolers’ active behaviors across the *entire* week; the same is true for minimizing this group’s sedentary behaviors. For instance, and in line with best practices for children in childcare [36], childcare providers may choose to implement additional active play-based activities/opportunities (inclusive of some that are teacher-led) on days where physical activity levels tend to be lower (*i.e.*, at the start and at the end of the week) to ensure active behaviors are being maintained over the course of the week. Childcare providers may also chose to provide additional periods for outdoor play mid-week as such opportunities have been linked to increased levels of physical activity among this cohort [19,20]. As evidenced by Tucker and colleagues’ [14] and van Zandvoort and colleagues’ work [15], additional training and education for parents/guardians regarding the importance of promoting activity in the home environment may be warranted to complement the positive activity messaging delivered by childcare staff. Such aids could be presented in the form of newsletters and handouts with activity ideas as well as offering workshops to parents based on active behaviors. Increased training for childcare staff may also prove useful in helping reinforce to these key individuals the importance of providing consistent and an increased number of physical activity opportunities to preschoolers during childcare hours.

From a policy perspective, instances to maximize physical activity opportunities during childcare hours should be explored and implemented. In fact, there is much research to substantiate the positive impact of physical activity-related policies on promoting active behaviors during childcare hours [13,37,38]. Childcare centers should aim to articulate in their policies specific time requirements in which enrolled preschoolers should participate in physical activity during care hours (or time restrictions in the case of sedentary behaviors). Furthermore, and as evidenced by previous work by members of this research team [13], childcare providers should be included in the process of developing both effective and feasible programs that target active and sedentary behaviors among preschoolers.

While the findings of the present study are interesting, additional research is required to confirm these results. A limitation of this study is that all children did not attend childcare on the same schedule. More specifically, some children attended full days every day, while others attended half-days every day, or full days on alternate days; this potentially may have impacted the activity trends observed in this study. Moreover, given the increasing attention paid to home- and/or family-based childcare programs, it may prove worthwhile to explore whether similar trends exist within this particular early learning setting as well. Finally, while the majority of this study’s data collection took place during the Spring, Summer, and Fall months (with the Winter period being avoided where able), it is still possible that weather may have played a factor in preschoolers’ access to outdoor playtime (which has been highly correlated with physical activity among this age group [38]). Future research should take care to account for weather and/or seasonality given its noted effects on preschoolers’ activity levels [39].

## 5. Conclusions

This was the first study conducted to explore whether preschoolers’ activity levels and sedentary time vary by day of the week during childcare hours. The findings from this paper suggest that preschoolers are most active mid-week, and as such, changes to daily programming and curricula within the childcare setting could be modified to better support active behaviors and limit sedentary time at both the start and end of the week. Future research is required to validate these findings.
